# Ferulic Acid Promotes Hypertrophic Growth of Fast Skeletal Muscle in Zebrafish Model

**DOI:** 10.3390/nu9101066

**Published:** 2017-09-26

**Authors:** Ya Wen, Hideki Ushio

**Affiliations:** Department of Aquatic Bioscience, Graduate School of Agricultural and Life Sciences, University of Tokyo, Tokyo 113-8657, Japan; zhxidtc@gmail.com

**Keywords:** ferulic acid, skeletal muscle mass, muscular hypertrophy, fast myofibers, sarcomeric unit, protein synthesis

## Abstract

As a widely distributed and natural existing antioxidant, ferulic acid and its functions have been extensively studied in recent decades. In the present study, hypertrophic growth of fast skeletal myofibers was observed in adult zebrafish after ferulic acid administration for 30 days, being reflected in increased body weight, body mass index (BMI), and muscle mass, along with an enlarged cross-sectional area of myofibers. qRT-PCR analyses demonstrated the up-regulation of relative mRNA expression levels of myogenic transcriptional factors (MyoD, myogenin and serum response factor (SRF)) and their target genes encoding sarcomeric unit proteins involved in muscular hypertrophy (skeletal alpha-actin, myosin heavy chain, tropomyosin, and troponin I). Western blot analyses detected a higher phosphorylated level of zTOR (zebrafish target of rapamycin), p70S6K, and 4E-BP1, which suggests an enhanced translation efficiency and protein synthesis capacity of fast skeletal muscle myofibers. These changes in transcription and translation finally converge and lead to higher protein contents in myofibers, as confirmed by elevated levels of myosin heavy chain (MyHC), and an increased muscle mass. To the best of our knowledge, these findings have been reported for the first time in vivo and suggest potential applications of ferulic acid as functional food additives and dietary supplements owing to its ability to promote muscle growth.

## 1. Introduction

Ferulic acid (FA) is a ubiquitous hydroxycinnamic acid derivative in the catalog of polyphenols [1]. It is also named 4-hydroxy-3-methoxycinnamic acid according to the presence of two motifs: the 4-hydroxyl and 3-methoxy groups on the phenolic nucleus, and the adjacent unsaturated carbon-carbon double bond on the extended side chain, which are highly related to its chemical properties [2]. Ferulic acid is widely distributed in the plant kingdom, including vegetables, fruits, and cereal grains in both free and bound forms [3,4,5]. It is generally found in the primary cell wall in the covalently conjugated form esterified with polysaccharides, glycoproteins, polyamines, lignin, and hydroxyl acids [6].

As a naturally existing antioxidant, ferulic acid has been a hot topic of research during recent years, especially in the field of human diseases and health [7]. There are many studies using ferulic acid against inflammation [8,9], cancer [10], cardiovascular disease [11], diabetes [12], and Alzheimer’s disease [13]. It is being considered a promising drug to lower cholesterol levels [14] and to target hyperglycemia [15]; it is also used as a food preservative and antioxidant supplement [16] in the food industry; and, it is also added in skin lotions and sunscreens as a UV protectant [17]. However, the effect of ferulic acid on the growth of skeletal muscle has hardly ever been reported in scientific literature.

Skeletal muscle is a plastic tissue playing essential roles in locomotion and energy metabolism [18]. In adults, the main pattern of skeletal muscle growth is referred to as muscular hypertrophy, which is characterized by an increase in muscle mass and an enlarged cross-sectional area due to the growth of myofibers [19]. The myofiber is the basic unit of the skeletal muscle and contains bundles of myofibrils, each of which is composed of sarcomeric units [20]. In adult fast skeletal muscle, the growth process of myofibers involves the coordinated expression of numerous muscle-specific genes, especially those encoding sarcomeric unit proteins, under the regulation of a series of transcription factors. MyoD and myogenin are myogenic regulatory factors (MRFs) responsible for myogenesis, and have been reported to positively regulate the gene expression of fast myosin heavy chain [21] and troponin I [22] in mice. Serum response factor (SRF), a ubiquitous transcription factor, also contributes to hypertrophic growth of fast skeletal muscle by enhancing the expression of alpha-actin gene as reported in chickens [23]. Additionally, mRNA translation and protein synthesis are initiated by the activation of the mTOR/p70S6K/4E-BP1 signaling pathway [24], which is a crucial regulator of muscular hypertrophy [25].

Zebrafish have emerged as one of the most acceptable research models in multidisciplinary fields, including development, physiology, and genetics because of the ease in maintaining and breeding the fish, the short culturing period due to a high reproductive capacity, and the availability of a comprehensive genomic background [26]. As vertebrates, zebrafish possess many advantages as a model organism, including structural similarities with humans, an equivalent for almost every human organ, and similar cellular and molecular pathways. As claimed by a recent genome study, up to 70% of protein-coding genes in human beings have counterparts in zebrafish, and the percentage of genes associated with human diseases has increased to up to 84% [27]. In the field of skeletal muscle research, zebrafish has been instrumental in key contributions to the understanding of skeletal muscle development [28], muscular disorders [29], and muscular hypertrophy [30], due to the numerous molecular features of skeletal muscle shared between zebrafish and mammalians, including a conserved transcriptional network regulating myogenesis, as well as similar histological and ultrastructural features [31].

In this study, zebrafish were used as the experimental model for investigating the effect of ferulic acid on skeletal muscle growth. Physiology index measurement and morphometric analyses were performed to identify the phenotypes in adult zebrafish after ferulic acid administration. To further understand the molecular mechanisms underlying the observed phenotypes, gene expression related to muscular hypertrophy and intracellular signaling pathways related to protein synthesis were examined.

## 2. Materials and Methods

### 2.1. Pre-Culturing of Zebrafish

Zebrafish (wild type, AB line) aged around three months post-fertilization were purchased from Renovo Science (Tokyo, Japan). All zebrafish were kept at 28 °C under a 14 h light: 10 h dark cycle, and water conditions of environmental quality were maintained according to *The Zebrafish Book* [32]. Zebrafish were allowed to adjust to the new environment for two weeks. Considering the influence of sexuality and complex situations such as the unsynchronized pregnancy status of female zebrafish, only male zebrafish were used for investigations in the present study.

### 2.2. Preparation of Experimental Feeds

A commercial feed (Nippai, Yokohama, Japan), consisting of 47% protein, 12% fat, 3% crude fiber, and 12% ash, was used. The commercial feed was ground into powder and mixed with 20 mg (*E*)-ferulic acid crystal (Wako, Tokyo, Japan) that was dissolved in 15 mL ddH_2_O at 95 °C, to prepare FA-containing feed at a concentration of 20 mg ferulic acid per 100 g commercial feed. Feed dough was made by repetitive rubbing and kneading. Subsequently, the dough was passed through a sieve (Testing sieve, Tokyo Screen Co., LTD., Tokyo, Japan) with an aperture diameter of 850 μm. After being frozen at −80 °C and subjected to lyophilization (EYELA, Tokyo, Japan), the resulting small pellets were adjusted into a suitable size for consumption by zebrafish by passing through sieves several times and were stored at 4 °C until use. Negative control feed (100 g commercial feed) was prepared by passing through the similar treatment without addition of ferulic acid.

### 2.3. Feeding Experiments

After acclimation, male zebrafish with a similar body index (body weight and length) were chosen as one population. Zebrafish in the population were allocated into two dietary groups: normal feeding group as a negative control (NC) and ferulic acid feeding group as an experimental group (FA). The fish were maintained at a density of five individuals per 2 L water tank. Each tank of fish was fed with 500 mg of experimental feeds every day for 30 days. Three populations with different initial body indices were under investigation in the present study.

### 2.4. Measurement of Body Index

Body weight and length of zebrafish from the three populations were measured on designated days 0, 10, 20, and 30 throughout this study. Before measurements, zebrafish were anesthetized with 2-phenoxyethanol (Wako, Tokyo, Japan) at 1:1000 dilution for 15 s. Total body length was measured from the head to the end of the body. Once the measurement was finished, zebrafish were put back into fresh water. Body mass index (BMI) was calculated as weight/length^2^.

### 2.5. Computed Tomography Measurement of Muscle Mass

A Computed Tomography (CT) scan was carried out on the final day of the feeding schedule. Zebrafish were fixed in a stretched position on a sample holder with diameter of 24 mm after being anesthetized with 2-phenoxyethanol at 1:1000 dilution and then scanned using a LaTheta^TM^ in vivo Micro-CT scanner (Hitachi Aloka Medical, Tokyo, Japan) using the following parameters: tube voltage, 80 kV; tube current, 0.5 mA; axial field of view (FOV); slice at every 2 mm; and, a total scan time of approximately 10 min. Quantitative assessment of muscle mass was performed with a LaTheta software (version 3.00, Hitachi Aloka Medical, Ltd., Tokyo, Japan).

### 2.6. Morphometric Analyses of Myofibers in Fast Skeletal Muscle

A fast skeletal muscle filet of 2 mm thickness of zebrafish was dissected at three quarters’ body length and immediately frozen. After placing the frozen samples in Tissue-Tek^®^ O.C.T compound embedding medium (SAKURA, Tokyo, Japan) at −25 °C, serial transverse sections of 8 μm thickness were obtained with Tissue-Tek^®^ Cryo_3_^®^ Cryostat (SAKURA, Tokyo, Japan). Slices were observed under a Keyence BZ-9000 digital microscope (KEYENCE, Osaka, Japan). The cross-sectional area of myofibers was measured using ImageJ (US National Institute of Health, available at http://rsb.info.nih.gov/ij/). In each muscle sample, 480 myofibers were analyzed. Data were processed using R language to make the histograms of fiber cross-sectional area (FCSA) illustrating the percentage frequencies of myofibers grouped in intervals of 500 μm^2^. To obtain the overlapped curves in the histograms, a dynamic fitting by log-normal regression was performed for zebrafish of each group (NC and FA group).

### 2.7. RNA Extraction and Quantitative Real-Time PCR

Dorsal epaxial fast skeletal muscle was dissected 1.5 h after feeding on the final day of the feeding experiment, was immediately immersed in liquid nitrogen, and then stored at −80 °C. Before RNA extraction, muscle tissues stored at −80 °C were subjected to homogenization with QIAzol^®^ Lysis Reagent (QIAGEN K.K., Tokyo, Japan). Total RNA was extracted using the RNeasy Mini kit (QIAGEN K.K., Tokyo, Japan) followed by qualification using NanoPhotometer^®^ (IMPLEN, München, Germany). cDNA was then synthesized using the PrimeScript^TM^ RT reagent Kit with gDNA Eraser (Perfect Real Time; TaKaRa, Tokyo, Japan). Quantitative real-time PCR was performed using Applied Biosystem 7300 Real-Time PCR System (Thermo Fisher Scientific Japan, Tokyo, Japan) with SYBR *Premix Ex Taq*^TM^ II (Tli RNaseH Plus; TaKaRa, Tokyo, Japan) following the manufacturer’s instructions. Primers designed for genes of interest are described in detail in Table 1. The reaction conditions were as follows: an initial step at 95 °C for 30 s, followed by 40 cycles of 95 °C for 5 s, and 60 °C for 31 s. The melt curve was obtained as follow: 95 °C for 15 s, followed by 60 °C for 60 s, and a reaction-termination at 95 °C for 15 s. The relative mRNA expression levels were normalized using *ribosomal protein L8* (*rpl8*) gene as an internal control [33].

### 2.8. Western Blot Analyses

Protein was extracted from fast skeletal muscle tissues in the dorsal part using 1× RIPA lysis buffer (#9806, Cell Signaling Technology Japan, Tokyo, Japan) with a protease inhibitor cocktail (Thermo Fisher Scientific Japan, Tokyo, Japan). After the quantification of proteins using the Pierce^TM^ BCA Protein Assay Kit (Thermo Fisher Scientific Japan, Tokyo, Japan), protein concentrations were adjusted to equal amounts before being subjected to SDS-PAGE and transferred onto a 0.22-μm polyvinylidene difluoride (PVDF) membrane (Merck Millipore, Bedford, MA, USA). Membranes were then blocked in 1× TBST (1× tris-buffered saline solution containing 5% bovine serum (BD, Franklin Lakes, NJ, USA) at room temperature for 1 h. The membranes were then incubated with primary antibodies of the proteins concerned at 4 °C overnight, respectively. Antibodies used in the present study were as follows: mTOR antibody (#2972), Phospho-mTOR (Ser2448) antibody (#2971), p70S6 kinase antibody (#9202), Phospho-p70S6 kinase (Ser371) antibody (#9208), 4E-BP1 antibody (#9452), and Phospo-4E-BP1 (Thr37/46) antibody (#2855) (Cell Signaling Technology Japan, Tokyo, Japan, 1:500); Myosin (Skeletal, Fast; Product No. M4276, Sigma-Aldrich, 1:500); GAPDH (D16H11) XP^®^ rabbit mAb (#5174, Cell Signaling Technology Japan, Tokyo, Japan, 1:1000). After being washed three times for 5 min each with 1× TBST at room temperature, membranes were then incubated with a secondary antibody Alexa Fluor 680 (Thermo Fisher Scientific Japan, Tokyo, Japan, 1:10,000) at room temperature for 1 h. Membranes were then washed with 1× TBST for 5 min × 3 times at room temperature before taking photos. ODYSSEY^®^ Fc Imaging System (LI-COR Biosciences, Lincoln, NE, USA) was used for scanning and detection of bands. The relative protein level in comparison to GAPDH was calculated by software Image Studio 2.0 (LI-COR Biosciences, Lincoln, NE, USA).

### 2.9. Statistical Analyses

All data were presented as mean ± SD. Differences between the two groups were examined by a one-tailed t-test for statistical significance. For multiple comparisons of body weight and BMI between the two groups with 4-time points, we performed a two-way ANOVA analysis, followed by a Bonferroni-Dunn multiple comparison using software GraphPad Prism 5.0 (GraphPad Software Inc., San Diego, CA, USA). *p* < 0.05 was considered to denote statistical significance.

## 3. Results

### 3.1. Body Weight and BMI Were Increased in the FA Group

After 30 days of the feeding experiment, somatic growth was observed in all of the groups. For each population, there was an increase of both body weight and length in the FA group as compared to the NC group. The specific data for each group in the population are as follows.

**Figure 1:**
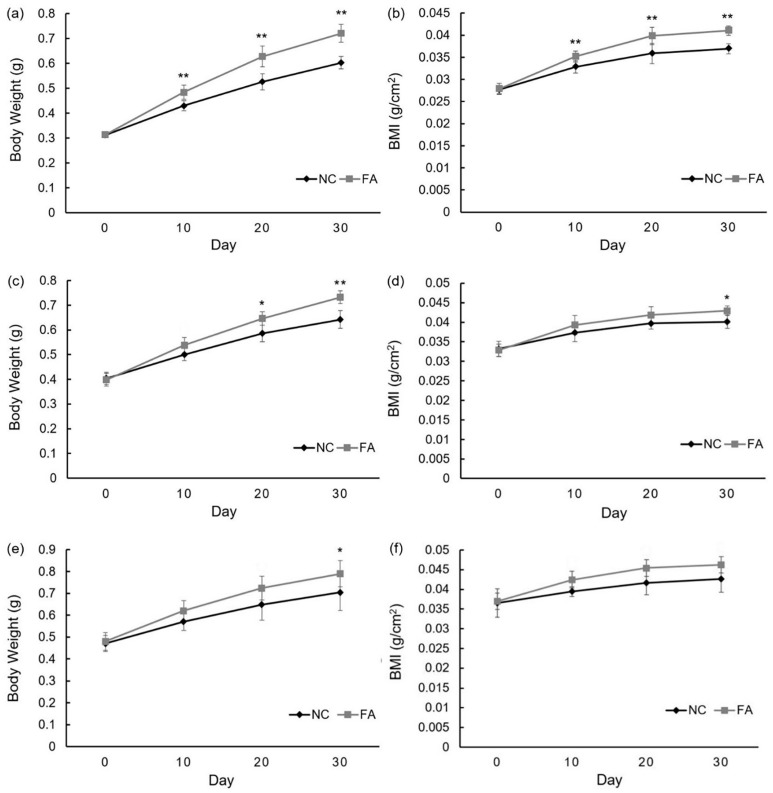
Effects of ferulic acid on body weight and body mass index of adult zebrafish. Population 1: (**a**) average body weight of each group; (**b**) average BMI of each group throughout feeding experiment; Population 2: (**c**) average body weight of each group; (**d**) average BMI of each group throughout feeding experiment; Population 3: (**e**) average body weight of each group; (**f**) average BMI of each group throughout the feeding experiment; NC: negative control group; FA: ferulic acid administered group. Data are expressed as mean ± SD (*n* = 10, for each group from Population 1; *n* = 5, per group from Population 2 and 3), * *p* < 0.05, ** *p* < 0.01 compared with the NC group.

In Population 1, the average body weight of the NC group increased from 0.312 ± 0.009 g to 0.603 ± 0.025 g, whereas the average BMI increased from 0.028 ± 0.001 to 0.037 ± 0.001. By contrast, the average body weight of the FA group increased from 0.314 ± 0.008 g to 0.721 ± 0.036 g, whereas the average BMI increased from 0.028 ± 0.001 to 0.041 ± 0.001. By the end of the feeding experiment, zebrafish of the FA group gained body weight and length by 19.57% and 11.10%, respectively, as compared to the NC group (Figure 1a,b). In Population 2, the average body weight of the NC group increased from 0.404 ± 0.025 g to 0.642 ± 0.036 g, whereas the average BMI increased from 0.033 ± 0.002 to 0.040 ± 0.002. By contrast, the average body weight of the FA group increased from 0.398 ± 0.026 g to 0.732 ± 0.026 g, whereas the average BMI increasing from 0.033 ± 0.002 to 0.043 ± 0.001. By the end of the feeding experiment, zebrafish of the FA group gained body weight and BMI by 14.02% and 6.99%, respectively, when compared to the NC group (Figure 1c,d). In Population 3, the average body weight of the NC group increased from 0.470 ± 0.037 g to 0.702 ± 0.082 g, whereas the average BMI increased from 0.037 ± 0.004 to 0.043 ± 0.003. By contrast, the average body weight of the FA group increased from 0.480 ± 0.041 g to 0.790 ± 0.060 g, whereas the average BMI increased from 0.037 ± 0.001 to 0.046 ± 0.003. By the end of the feeding experiment, zebrafish of the FA group gained body weight and total length by 12.22% and 8.62%, respectively, as compared to the NC group (Figure 1e,f).

Differences in body weight and BMI showed up around day 10. This trend was maintained throughout the study. Among three populations, Population 1, with the lowest initial body weight and BMI, demonstrated the most significant trend.

### 3.2. Muscle Mass Was Increased in the FA Group

**Figure 2:**
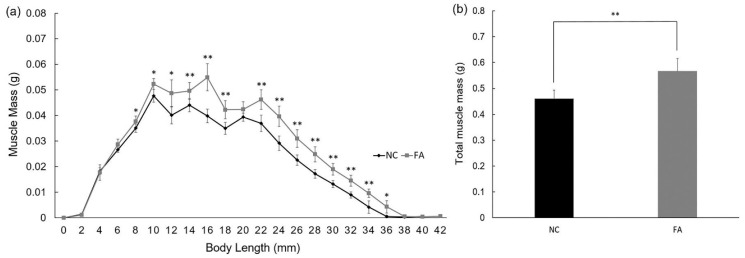
Effects of ferulic acid on muscle mass of adult zebrafish. (**a**) Muscle mass contained per slice in each group. Computed Tomography (CT) scan was taken every 2 mm from head to tail; (**b**) Average muscle mass in total for each group. Data are expressed as mean ± SD (*n* = 5 per group from Population 1), * *p* < 0.05, ** *p* < 0.01 compared with the NC group.

To analyze the source of weight and BMI gain, we conducted CT scans for skeletal muscle mass measurement using zebrafish from Population 1. There was a significant increase in the muscle mass content in most of the slices in the FA group when compared to the NC group within the body length range of 8 mm to 36 mm (Figure 2a). The cumulative increase finally led to a significantly higher amount of total muscle mass of zebrafish in the FA group (0.566 ± 0.050 g) relative to the NC group (0.460 ± 0.033 g; Figure 2b). The comparison of the FA group to the NC group showed that the relative increase in total muscle mass (0.105 g) accounted for 88.98% of the relative increase in body weight (0.118 g). Muscle mass peaked at 14 mm in the NC group, and at 16 mm in the FA group, demonstrating 2 mm of extra trunk growth in the FA group as compared to the NC group during this range of body length (Figure 2a).

### 3.3. Size of Fast Skeletal Myofibers Were Enlarged in the FA Group

**Figure 3:**
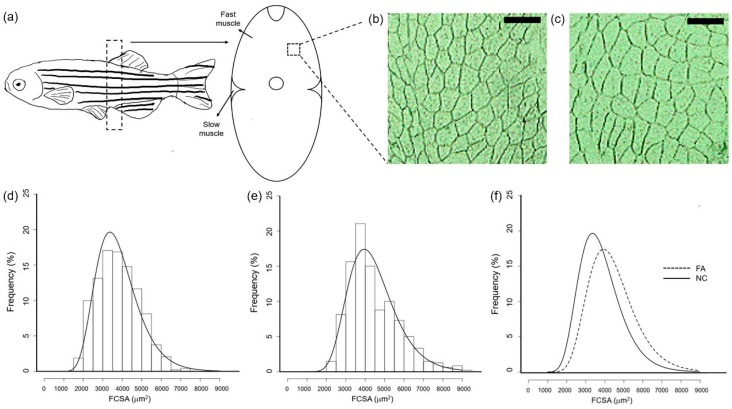
Effects of ferulic acid on the cross-sectional area of fast skeletal myofibers in adult zebrafish. (**a**) Image of fast skeletal muscle tissue used for cross section analysis. Frame with dotted line shows the position of filet dissection; Cross section of fast skeletal muscle of zebrafish from the (**b**) NC and (**c**) FA groups; Square with dotted line shows the area of (**b**,**c**). Bar represents 150 μm (**d**) Fiber cross-sectional area histograms for fast skeletal muscle of adult zebrafish in the NC group and (**e**) in the FA group; (**f**) Merged curves of (**d**,**e**). Samples of each group were from Population 3. FCSA: fiber cross-sectional areas (μm^2^) were grouped in intervals of 500 μm^2^. Curves represent a log-normal regression.

In order to evaluate the effect of ferulic acid administration on cellular characteristics of the muscle, frozen sections of fast skeletal muscle in the NC and FA groups were observed. Fast skeletal myofiber frequency distribution was analyzed for both groups, after fiber cross-sectional area (FCSA) measurement. There were more cell numbers with a larger size in the FA group (Figure 3c) when compared to the NC group (Figure 3b). As shown in Figure 3d–f, the log-normal regression curves were centered around higher FCSA values in the FA group (approximately 4000 μm^2^) than in the NC group (approximately 3500 μm^2^), and the regression curve of the FA group was significantly shifted to the right as compared to that of the NC group. In detail, zebrafish from the FA group presented a lower percentage of small fibers (FCSA < 4000 μm^2^) but a higher percentage of medium (with area between 4000 μm^2^ and 6000 μm^2^) and large fibers (FCSA > 6000 μm^2^) compared to zebrafish from the NC group.

### 3.4. Increase in Transcription Level of Genes Related to Muscular Hypertrophy in the FA Group

**Figure 4:**
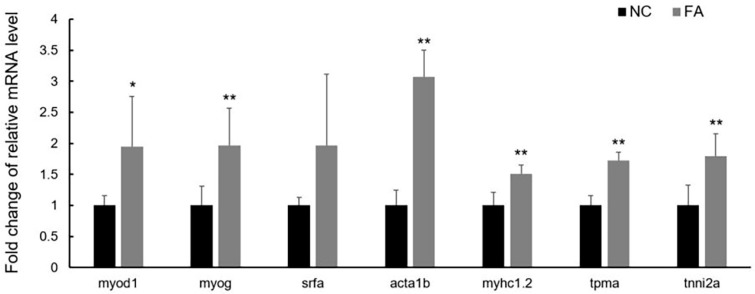
Effects of ferulic acid on gene transcription of fast skeletal muscle in adult zebrafish. Raw data were calculated in 2^ΔΔ−ct^ method. Relative mRNA expression level of genes in the FA group was shown as fold change after being normalized to the NC group. NC: negative control group; FA: ferulic acid administered group. Data is expressed as mean ± SD (*n* = 5, per group from Population 2), * *p* < 0.05, ** *p* < 0.01 when compared to the NC group.

To detect the molecular changes occurring in fast skeletal muscle of zebrafish after ferulic acid administration for 30 days, gene expression levels of transcription factors that play important roles in muscle hypertrophy were examined, including MyoD, myogenin, and SRF. The relative mRNA levels of *myod1* (1.950 ± 0.804; *p* = 0.028) and *myogenin* (1.963 ± 0.604; *p* = 0.009), suggest significant up-regulation in transcription of these genes in the FA group compared to the NC group, whereas the relative RNA level of *srfa* (1.968 ± 1.148; *p* = 0.066), suggests a similar trend (Figure 4). As the targets of above transcription factors, genes encoding sarcomeric unit proteins, such as alpha-actin, myosin heavy chain, tropomyosin, and troponin I, were also examined. The relative mRNA levels of *acta1b* (3.074 ± 0.429; *p* = 2.86 × 10^−5^), *tropomyosin* (1.721 ± 0.136; *p* = 2.65 × 10^−5^), *myhc1.2* (1.508 ± 0.142; *p* = 0.001), and *tnni2a* (1.795 ± 0.363; *p* = 0.003), suggest significantly elevated transcription of these genes in the FA group when compared to the NC group (Figure 4).

### 3.5. Translation Efficiency Was Enhanced and Protein Synthesis of Sarcomeric Unit Was Elevated in the FA Group

**Figure 5:**
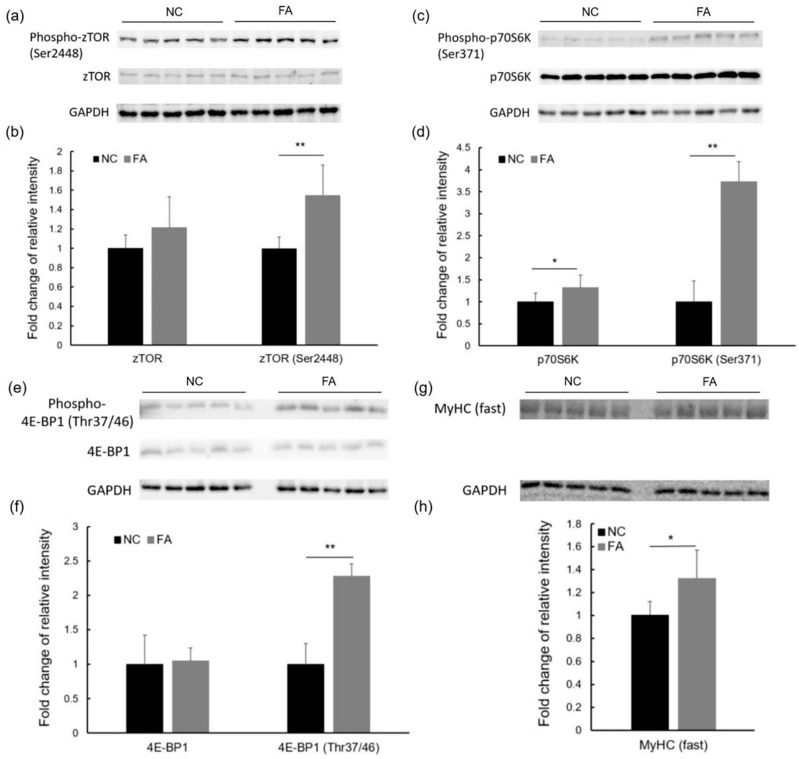
Effects of ferulic acid on protein synthesis of fast skeletal myofibers in adult zebrafish. (**a**) Western blot and (**b**) fold change of relative intensity of zTOR, Phospho-zTOR (Ser2448); (**c**) Western blot and (**d**) fold change of relative intensity of p70S6K, Phospho-p70S6K (Ser371); (**e**) Western blot and (**f**) fold change of relative intensity of 4E-BP1, Phospho-4E-BP1(Thr37/46); and, (**g**) Western blot and (**h**) fold change of relative intensity of myosin heavy chain, MyHC (fast). The relative intensity of all proteins was calculated after normalizing with GAPDH. Data from the FA group are shown as fold changes after being normalized to the NC group. NC: negative control group; and, FA: ferulic acid administered group. Data are expressed as mean ± SD (*n* = 5, per group from population 2), * *p* < 0.05, ** *p* < 0.01 compared to the NC group.

The increase in protein synthesis, which can be regulated by the major anabolic pathway, mTOR signaling, is a key feature of muscular hypertrophy [24,25]. As the most studied downstream effectors of mTOR signaling, p70S6K and 4E-BP1 play important roles in the initiation of mRNA translation. To confirm the activation of the protein synthesis pathway, we determined the phosphorylation levels of zebrafish target of rapamycin (zTOR), the homolog of mTOR [34], p70S6K, and 4E-BP1 by Western blot analysis. As shown in Figure 5a–f, the relative phosphorylation levels of zTOR, p70S6K, and 4E-BP1 normalized to GAPDH as an internal control, were significantly increased in the FA group as compared to the NC group. This increase is described by the following fold changes: Phospho-zTOR (Ser2448; 1.55 ± 0.31; *p* = 0.007), Phospho-p70S6K (Ser371; 3.72 ± 0.46; *p* = 7.89 × 10^−6^), and Phospho-4E-BP1 (Thr37/46; 2.28 ± 0.18; *p* = 1.11 × 10^−4^). In addition, a trend was observed in the relative protein expression level of zTOR (1.21 ± 0.32; *p* = 0.11) after ferulic acid administration. A significant increase in the relative protein expression level of p70S6K was seen in the FA group (1.32 ± 0.29; *p* = 0.041) when compared to the NC group. As a result of the higher gene transcription level of myosin heavy chain and a higher mRNA translation efficiency, the protein abundance of myosin heavy chain was significantly increased in the FA group as compared to the NC group as shown in fold change: MyHC (1.32 ± 0.25; *p* = 0.020; Figure 5g,h).

## 4. Discussion

In the present study, we used zebrafish as the experimental subject to explore the potential effects of ferulic acid. The observed higher body weight and BMI in zebrafish from the FA group over that from the NC group in feeding experiments provided the first clue in our study. Body composition analyses carried out by a CT scan on the whole body suggested that increased muscle mass was the major source of weight gain. The enlarged size of fast skeletal myofibers, as demonstrated in morphometric analyses, provides evidence for the hypertrophic phenotype of fast skeletal muscle in adult zebrafish after ferulic acid administration for 30 days. These observations indicate that ferulic acid promotes the growth of adult zebrafish, increases muscle mass, and induces hypertrophic growth of fast skeletal muscle.

The skeletal muscle specific transcription factors MyoD, myogenin, and the ubiquitously expressed SRF, are considered as master regulators in the hypertrophic growth of adult skeletal muscle. As members of MRFs, MyoD and myogenin are involved in different stages of myogenesis: MyoD is vital for the commitment of myogenic precursor cells to myoblasts through activation, proliferation, and differentiation, whereas myogenin is responsible for the subsequent maturation and fusion of myoblasts to pre-existing myofibers, thereby contributing to hypertrophic growth [35]. There is an increase in transcription of MyoD and myogenin genes during Insulin-like growth factor 1 (IGF-1) overexpression-induced hypertrophy in mice [36]. SRF is also a key factor controlling skeletal muscle hypertrophy due to its participation in all the above-mentioned stages of myogenesis [37]. As reported in other studies, there is an increase of SRF expression during overload-induced hypertrophy in rats [38] and a decrease of SRF expression during disuse-induced muscle atrophy in humans [39]. In our study, higher transcriptional levels of MyoD, myogenin, and SRF genes were detected in the FA group as compared to the NC group, which suggests hypertrophic growth of fast skeletal muscle after ferulic acid administration. 

Furthermore, these transcription factors are believed to positively regulate the transcription of muscle-specific genes. MyoD and myogenin have been reported to activate the MyHC IIb (myosin heavy chain, type IIb) promoter in mice through the binding of their highly conserved basic helix-loop-helix (bHLH) motifs to the E-box (5′-CANNTG-3′) present in the promoter region [40]; the interaction of MyoD, myogenin, and the troponin I internal regulatory element (TnI IRE) is a prerequisite for troponin I gene transcription [22]; SRF has been reported to enhance the transcription of skeletal alpha-actin and tropomyosin gene after recognition and binding to the CArG box (5′-CC[A/T]_6_GG-3′) present in the *cis*-regulatory region [41]. In our study, we detected higher transcription levels of genes encoding sarcomeric unit proteins in the FA group in comparison with the NC group. The increase in expression of sarcomeric unit proteins would contribute to an enlarged myofiber size, since they are the major components of the myofiber. 

In vivo, the mTOR/p70S6K/4E-BP1 signaling pathway is another crucial regulator of muscular hypertrophy. Existing as the multi-protein signaling complexes of mTOR, mTORC1 responds to various hypertrophic stimuli, such as mechanical loading, feeding, and growth factors [24,25,42]. The activated mTORC1 has been reported to not only increase translation efficiency, leading to protein synthesis, but also to inhibit autophagy, leading to protein retention [43]. Upon the arrival of the phosphorylation cascade at mTORC1, it is activated and in turn phosphorylates and activates p70S6K, but inactivates 4E-BP1. Phosphorylated p70S6K then phosphorylates S6, the protein of the 40S ribosomal subunits [44]. Meanwhile, phosphorylated 4E-BP1 relieves the inhibition of the translation initiator eIF4E as a result of dissociation from each other. Subsequently, the detached eIF4E binds with other factors to form the eIF4F complex and recruits the phosphorylated S6 to 5′ end of mRNA for translation initiation [25,42]. Thus, the activation of mTOR/p70S6K/4E-BP1 pathway results in the initiation of translation and protein synthesis. In our study, we found a relatively higher level of phosphorylation of zebrafish target of rapamycin (zTOR) at residue Ser2448, p70S6K at residue Ser371, and 4E-BP1 at residue Thr 37/46, in the FA group over the NC group, which suggests an enhanced mRNA translation efficiency and an increased overall rate of protein synthesis after ferulic acid administration. Although autophagic systems were not evaluated in this study, the effect of ferulic acid on them will be investigated and discussed in our future work, considering the contribution of autophagic systems to muscle mass regulation.

The combination of higher transcription levels of genes encoding sarcomeric unit proteins, higher translation efficiency, and enhanced protein synthesis capacity, can result in a higher amount of proteins in hypertrophic muscle cells. The higher protein level of myosin heavy chain (MyHC), a subunit of myosin, detected in fast skeletal muscle of zebrafish in the FA group supports this hypothesis. These changes occurring on molecular levels finally converge and lead to the increase of protein contents in myofibers, enlarged fiber size, and gain in muscle mass.

Currently, there is an increasing demand for muscle growth and building to support health and a higher quality of life. Gain in muscle mass improves the appearance and function of the body, as well as improving the metabolism of the whole body [18,45]. The process of ageing may induce the degenerative loss of skeletal muscle mass, referred to as sarcopenia, and subsequently decreased quality and strength, which is accompanied by a smaller myofiber size of fast skeletal muscle [46]. Therefore, due to its natural, ubiquitous occurrence, and low toxicity, ferulic acid could be a candidate additive to functional foods and dietary supplements for the purpose of muscle growth.

## Figures and Tables

**Table 1:** Primer pair sequences and accession numbers of the genes of interest.

Gene	Accession Number	Sequences of Primers (5′–3′)
*myod1*	NM_131262.2	F: CGAGAAGACGGAACAGCTATG
		R: CGGTGTCACTCAGGACAGATC
*myog*	NM_131006.1	F: CTGGGGTGTCGTCCTCTAGT
		R: TCCCGTTATGCTGTCCACTAT
*srfa*	NM_001110526.1	F: TTGACAACAAGCTGAGGAGATAC
		R: AAGTCTGGATCAGGGCTTTAC
*acta1b*	NM_214784.2	F: TGTGTGACGACGACGAGACTAC
		R: TGGGATATTTCAGAGTGAGGATAC
*myhc1.2*	ENSDARG00000067995	F: GTGGTTGATGACAAAGAGCTGTA
		R: GCACAGAGGGTTCATTGAGAT
*tpma*	AF180892.1	F: GAGGCTGATCGCAAGTATGA
		R: GACCTTGATCTCCTCCTCATATT
*tnni2a*	NM_001009901.2	F: CAAGGTTGATGAGGAGAGATATG
		R: TCCTTGACCTCCTTCTTGACTT
*rpl8*	NM_200713.1	F: AATCCACACCGGCCAG
		R: GCCAACGGGAAGCACA

*myod1*: myogenic differentiation 1; *myog*: myogenin; *srfa*: serum response factor alpha; *acta1b*: actin alpha 1b, skeletal muscle; *tpma*: alpha-tropomyosin; *myhc1.2*: myosin heavy polypeptide 1.2, fast skeletal muscle specific; *tnni2a*: troponin I type 2a, fast skeletal muscle specific; *rpl8*: ribosomal protein L8, as an internal control.
